# Intracardiac fluid dynamic analysis: available techniques and novel clinical applications

**DOI:** 10.1186/s12872-024-04371-3

**Published:** 2024-12-19

**Authors:** Isabella Leo, Angelica Cersosimo, Jessica Ielapi, Jolanda Sabatino, Federico Sicilia, Antonio Strangio, Stefano Figliozzi, Daniele Torella, Salvatore De Rosa

**Affiliations:** 1 Department of Experimental and Clinical Medicine, Magna Graecia University, Catanzaro, Italy Viale Europa, 1, 88100; 2 IRCCS Humanitas Research Hospital, Via Manzoni 56, 20089 Rozzano, Milan, Italy; 3 Department of Medical and Surgical Sciences, Magna Graecia University, Viale Europa, 1, 88100 Catanzaro, Italy; 4 University of Naples Federico II, Via Pansini, 80131 Napoli, Italy; 5 School of Biomedical Engineering & Imaging Sciences, King’s College London, London, UK

**Keywords:** Fluid dynamics, Vortex, Cardiac mechanics, Phase contrast magnetic resonance imaging, 4d Flow

## Abstract

There is a growing interest in the potential use of intracardiac fluid dynamic analysis to better understand cardiac mechanics and identify novel imaging biomarkers of cardiovascular disease. Abnormalities of vortex formation and shape may in fact play a critical role in cardiac function, affecting both efficiency and myocardial workload. Recent advances in imaging technologies have significantly improved our ability to analyze these dynamic flow patterns in vivo, offering new insights into both normal and pathological cardiac conditions. This review will provide a comprehensive overview of the available imaging techniques for intracardiac fluid dynamics analysis, highlighting their strengths and limitations. By synthesizing the current knowledge in this evolving field, the paper aims to underscore the importance of advanced fluid dynamic analysis in contemporary cardiology and to identify future directions for research and clinical practice.

## Introduction

Blood flowing into the heart chambers interacts with the myocardium, valves and other cardiac structures, undergoing continuous directional shifts. In detail, vortical patterns have been observed during different phases of the cardiac cycle, with specific geometry and anatomical locations that are strictly related to cardiac function and myocardial efficiency. Vortices within the great vessels were firstly described by Leonardo Da Vinci [1], and are known as key elements during cardiac structure embryonic development [2]. Among the multiple fluid dynamic parameters, there is a recent growing interest in vortex analysis given their potential role as surrogate of cardiac efficiency and markers of cardiac mechanics [3];abnormalities of these structures have been in fact related to cardiovascular (CV) disease and poor CV outcomes [3, 4]. Non-invasive evaluation of cardiac function is one of the most important steps during clinical evaluation and is usually based on standard transthoracic echocardiography (TTE) imaging parameters such as ejection fraction (EF); however, these parameters may be inaccurate in certain clinical settings and are often only late markers of CV disease [5]. There is therefore constant interest in developing and using novel techniques and parameters able to identify even subtle changes of myocardial function [5–8].

Understanding vortex formation and their changes in physiological as well as pathological conditions may add a piece to this puzzle. The aim of this narrative review is to summarize the available evidence in the field, from the physiology behind vortex formation to the imaging techniques available for their assessment and their potential clinical applications.

### Understanding vortex: how do they form and what’s their contribution to cardiac mechanics?

At the beginning of diastole, the relaxation and untwisting of the left ventricular (LV) myocardium cause a reduction in intraventricular pressure that leads to the opening of the mitral valve (MV). As blood flows from the atrium through the MV, it strikes the edges of the valve leaflets and, due to the different velocity between the flow and the leaflets, starts to organize in shear layers [9, 10]. These layers are directed from the MV opening toward the apex, where they roll up, creating a pair of asymmetric vortices as a result of the different lengths of the leaflets [10–12].

**Fig. 1:**
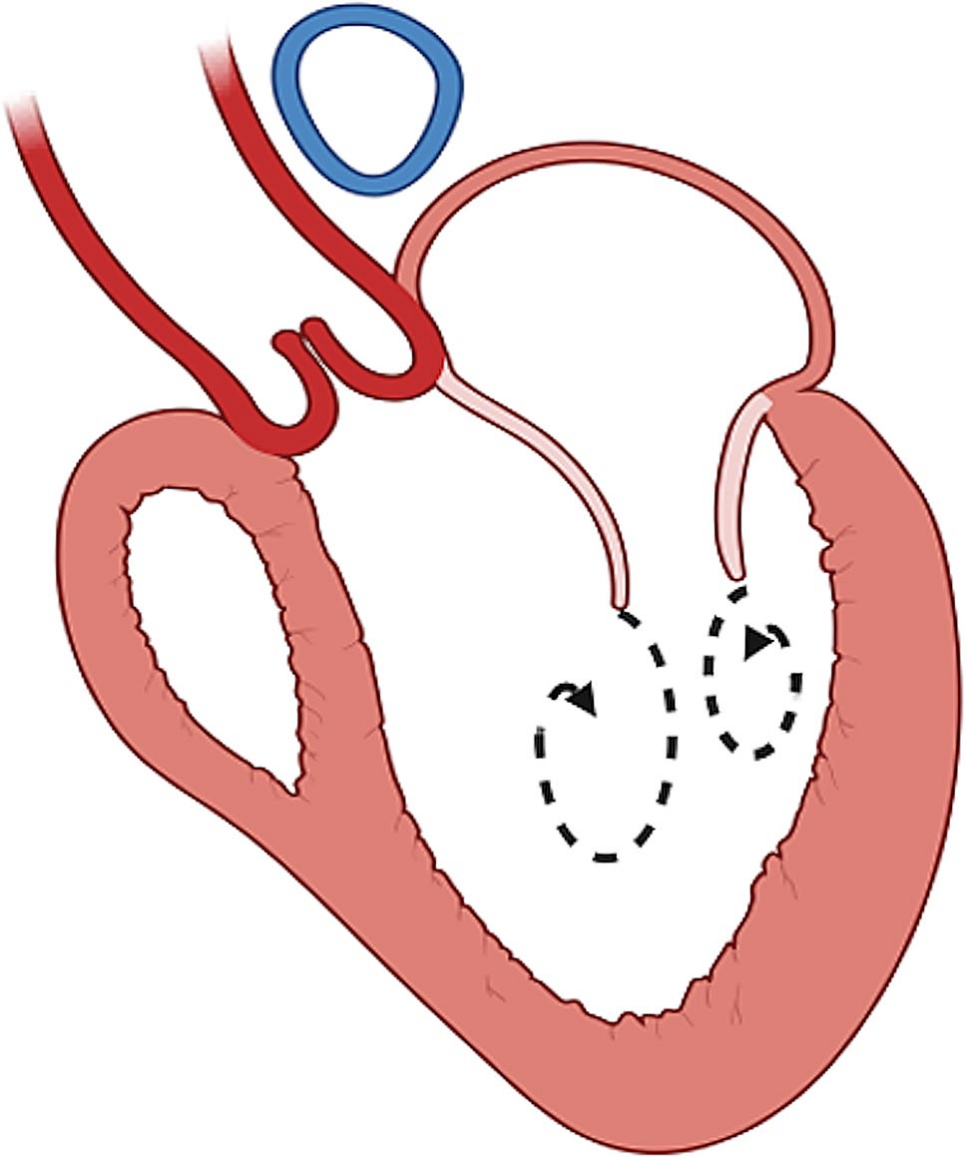
Vortex formation in the left ventricle. Two main vortices are identifiable in the left ventricle, one located at the anterior MV leaflet level and characterized by clockwise rotation, and another one distal to the posterior MV leaflet with counterclockwise rotation

This results in two clearly distinguishable vortices, one located at the anterior MV leaflet level and characterized by clockwise rotation, and another one distal to the posterior MV leaflet with counterclockwise rotation **(**Fig. 1**)** [12, 13]. During diastasis, the vortices remain in the LV cavity helping, with their rotation, to store kinetic energy (KE) [10]. Atrial contraction at the end of diastole can then generate a second, late, vortex located at a more basal level [14]. These vortices facilitate blood flow from the inlet to the outlet, thereby reducing the overall amount of energy needed during systolic contraction. Beyond qualitative assessment, several parameters can be used to quantitatively characterize these structures.

*Vorticity* measures the swirling motion of the fluid at a specific point, with the possibility to depict the intensity of rotation using a color gradient [10]. *Circulation* is instead the measure of the total vorticity within a specific region, and can be calculated using the line integral of the vortex vorticity in a specific region, for instance the entire LV [10, 15]. As already mentioned, large-scale vortices serve as a storage of KE, which is in part dissipated during the cardiac cycle due to viscous friction. The amount of KE dissipation can be also estimated and is inversely correlated with the overall efficiency of the system. The main direction of the flow identified as the angle between the flow momentum and a reference point represent the *flow force angle* [10]. Hemodynamic forces represent the force expressed by the entire blood volume to the surrounding boundary [16]. Usually, they are longitudinally oriented in a base-apex direction, while an increase in transverse forces has been observed in pathological conditions [4, 12].

Vortex ring development is often quantified using a dimensionless parameter called *vortex formation time* (VFT). VFT is a vortex duration property, calculated as the product of the time-averaged fluid velocity and the duration of ejection, divided by the orifice diameter. In healthy subjects, optimal VFT ranges between 3.3 and 5.5, aligning with results from in vitro studies [12]. Other vortex properties, related to vortex location and morphology are summarized in Table 1.

During systole, the blood ejected into the aorta is organized into vortices mainly within the sinuses of Valsalva, as a result of the high velocity and pulsatile nature of the flow and the curvature of the vessel itself [17]. Variations in the aorta’s shape, curvature, and diameter may all impact vortex formation [17, 18]. The role of these structures could be to facilitate a more rapid closure of the aortic valve and to support coronary blood flow [19].

Right ventricular fluid dynamics is gaining increasing interest. Vortex function in the RV seems to be similar to what described for the LV, acting as a facilitator in directing the blood flow toward the outflow tract and the pulmonary artery [20]. In detail, this mechanism seems to be easier in the right chambers, due to the physiological curvature of the basal and mid-ventricular RV segments [21]. An initially compact vortex ring is generated at the level of the tricuspid valve, but partially breaks in weak turbulent flow after collision with the interventricular septum [21].The remaining rotation gives rise during systole to a helical motion directed along the RV outflow tract [21]. It should be noted that the RV apex contributes less to overall RV contraction than the inflow and outflow, so it has been proposed that RV apex could play an important role in the maintenance of intracavitary circulation [22]. Fredriksson et al. suggested that this mechanism may facilitate smooth redirection and preservation of KE of high-velocity inflow blood towards the outflow tract, opposing to flow deceleration determined by apical trabeculations [21].

Atrial fluid dynamics have been primarily investigated in patients with atrial fibrillation (AF). Recent studies have shown that blood stasis, predicted by left atrial ejection fraction and left atrial retention ratio, is a strong predictor of stroke risk [23]. CFD simulations have demonstrated that blood stasis may be present even in the presence of sinus rhythm in patients with paroxysmal AF [23].Furthermore, assessment of left atrial fluid dynamics indicate that thrombogenic risk is not associated with the complex anatomy of the left atrial appendage. Slower flow, linked with high thrombogenic risk can also be observed in simpler anatomies [24]. A small CFD -based prospective study including five patients with AF who underwent left atrial plication during mitral valve surgery suggested that this intervention could reduce blood stasis more effectively than left atrial appendage resection [25].

**Table 1:** Main vortex properties and their definition

Vortex properties		
Parameter	Description	Formula
Vortex Area (VA)	VA is obtained tracing the outer ring of the main vortex at its maximum area, indexed to the LV (left ventricle) area. VA is correlated with LV geometric and volumetric parameters, and is therefore abnormal in cases of LV dysfunction.	VA = total VA/LV area
Vortex Width (VW)	VW is the horizontal (medio-lateral) diameter of the vortex in relation to LV transverse width, describing the lateral spread of the vortex. The width, along with the length, helps define the overall shape of the vortex, which is consistently wider than normal in cases of LV dysfunction. A wider vortex might indicate areas of flow separation or regurgitant flow in cases of valvular regurgitation.	VW = vortex width/LV width
Vortex Length (VL)	VL is the longitudinal (apico-basal) diameter of the vortex relative to LV length. This dimension represents the span of the vortex from its origin to its dissipation point, and can be an indicator of how long the vortex persists within the LV. VL is reduced in cases of LV dysfunction.	VL = vortex longitudinal length/LV length
Vortex Sphericity Index (VSI)	VSI is the ratio of vortex length to vortex width, allowing for definition of the vortex’s geometry and shape. This value is reduced in cases of LV dysfunction, where the vortex tends to be consistently wider, shorter, and consequently rounder than normal.	VSI = VL/VW
Vortex Depth (VD)	VD represents the distance of the vortex center from the base (mitral annulus), indexed to the long-axis LV length. It provides the vertical position of the vortex core within the LV. VD is expressed as a decimal between 0 and 1, approaching 1 when the vortex center is nearer the LV apex, and nearing 0 when it is closer to the LV base.	VD = distance of vortex center from LV base/LV long axis
Vortex Transversal position (VT)	VT provides the transverse position of the vortex core, indicating the distance of the vortex center from the posterior wall, indexed to the LV posteroseptal length.	VT = distance of vortex center from posterior wall/LV posteroseptal axis
Vortex Intensity (VI) or Strength (VS)	VI is the sum of the clockwise (CW) and counterclockwise (CCW) vortex circulation, calculated as the integral of vorticity within the vortex. It represents the intensity of the vortex’s rotational activity and is reduced in cases of LV dysfunction.	VS = CW circulation + CCW circulation
Relative Strength (RS)	RS represents the strength of the pulsatile component of vorticity in comparison to the average vorticity in the entire LV. It is defined as the total LV clockwise and counterclockwise vorticity strength of the first-order Fourier harmonic (the primary pulsatile contribution), indexed to the vortex strength of the zeroth-order Fourier harmonic (the steady contribution). This value is lower in patients with abnormal systolic function.	RS = total VS (first order)/total VS (zeroth order)
Vortex Relative Strength (VRS)	VRS represents the strength of the pulsatile component of a single vortex in comparison to the average vorticity across the whole LV circulation. It is calculated as the first-order Fourier harmonic of the vortex vorticity indexed to the total LV vorticity of the zeroth-order Fourier harmonic. This value is lower in patients with abnormal systolic function.	VRS = vortex VS (first order)/total VS (zeroeth order)
Vortex Pulsation Correlation (VPC)	VPC is the correlation between the steady and pulsatile components of the vortex within the LV and the vortex area, normalized by the square of the zeroth-order vorticity. It reflects vortex dynamics activity, with higher values indicating a coherent vortex with rhythmic alternation of formation and ejection. Reduced values indicate vortex stagnation and limited blood exchanges.	VPC = vortex VS correlation (first and zeroeth order) x vortex area/square total VS (zeroeth order)
Vortex Circulation (VC)	VC quantifies the intensity of the vortex (vorticity) over the main diastolic vortex, calculated as the integral of vorticity within the vortex, normalized by the total LV vorticity. Higher VC values indicate stronger vortices and may be associated with significant turbulence, suggesting pathologic conditions. This measurement can be applied to either the clockwise or counterclockwise vortices.	Circulation = vortex vorticity/total vorticity
Vortex formation time (VFT)	VFT measures the time required for vortex formation during a phase of the cardiac cycle, such as ventricular filling (diastole). It is calculated by dividing the length of the E-wave jet by the diameter of the mitral valve. VFT correlates with cardiac efficiency and represents an index of early diastolic filling in the LV, depending on both mitral geometry and trans-mitral flow.	VFT = VTI_E−wave_/D
Kinetic energy (KE)	KE measures the energy associated with the rotational motion of the flow within the vortex in LV area or volume. Higher KE values may suggest excessive turbulence, which can impose mechanical stress on the heart walls.	KE(x, y,t) = 1⁄2r(vx2 + vy2), where *r* is the density (*r* = 1,050 kg/m3)
Kinetic Energy Dissipation (KED)	KED represents the KE dissipated within the LV throughout the cardiac cycle, serving as an indirect measure of efficiency. Lower KED values indicate greater system efficiency. KED is increased in post-MI patients with LVEF > 50%, dilated cardiomyopathy (DCM)and Fontan circulation, and decreased in patients with ischemic LV systolic dysfunction (LVDF) and Tetralogy of Fallot (TOF).	Integral over the heartbeat of the rate of energy dissipation
Kinetic Energy Fluctuation (KEF)	KEF is the standard deviation of KE, normalized by its corresponding average value, representing the intensity of velocity fluctuation in perpendicular directions. Turbulent kinetic energy is increased in DCM patients, with values rising as the size of LV increases.	KEF = KE-SD/KE-average
Energy Loss Index (ELI)	ELI measures energy dissipation within the LV relative to the initially available KE. It represents the total energy lost as KE and viscous friction. Energy loss increases with cardiovascular and systemic diseases involving cardiovascular dysfunction.	ELI = Δ KE/KEinflow
Flow Force Angle (FFA)	The dominant direction of flow momentum is identified by an average angle, which lies between longitudinal and transversal forces. Typically, this angle ranges from 0° for longitudinal flow momentum to 90° for transversal flow momentum.	sin^2φ^ = Σ F x sin^2^ө/ Σ F

### Echocardiography

**Fig. 2:**
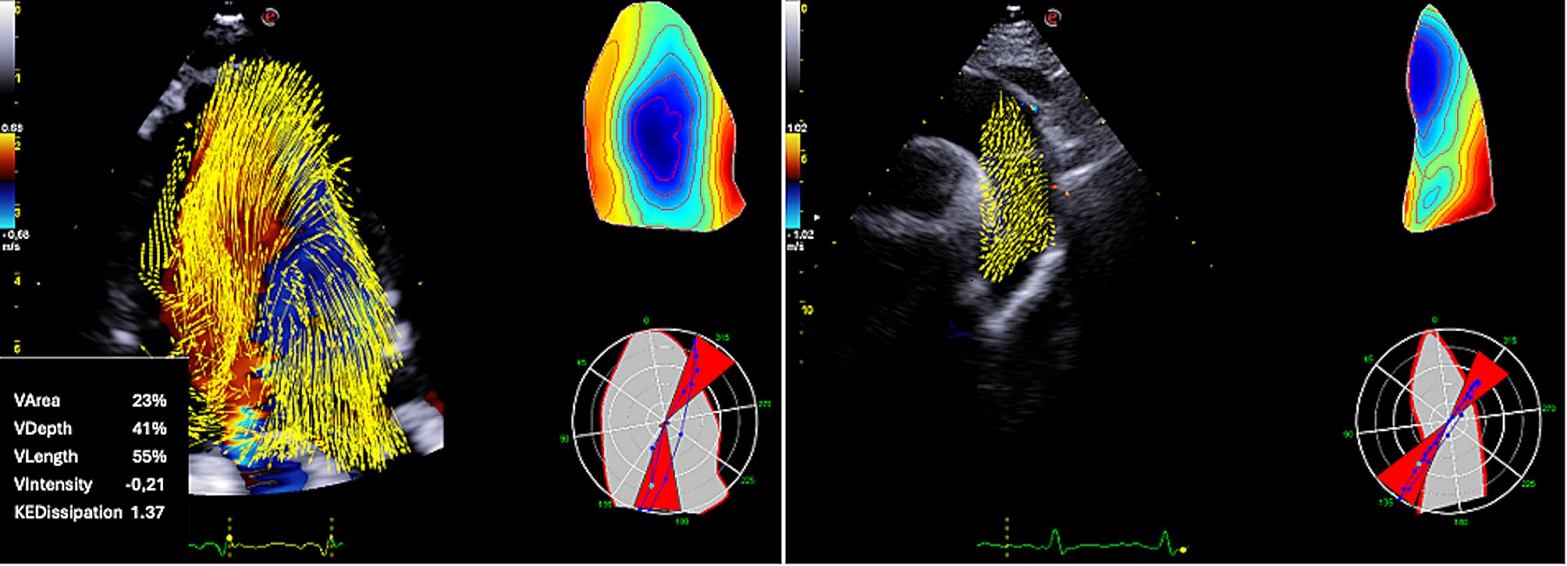
Intracardiac flow analysis using vector flow mapping. Fluid dynamic evaluation using the HyperDoppler Software in a patient with interventricular septal defect (on the left). The software allows both qualitative (polar and vector maps) and quantitative assessment of main vortex parameters. On the right, evaluation of the aortic arch using the same technique

Echocardiography is often the first-line imaging modality used to study blood flow within the vessels and the heart chambers [26, 27]. However, standard TTE primarily captures the longitudinal component of blood velocity, lacking information about the vortical organization and lateral direction of flow [28, 29]. To address this limitation, advanced echocardiographic techniques have been developed to provide a non-invasive assessment of intracardiac fluid dynamics. Vector Flow Mapping (VFM) combines Doppler data with speckle-tracking echocardiography (STE) to create a detailed map of intracardiac flow patterns and compute flow velocity vectors. Images are usually acquired in the apical three chamber view, using a 5–8 Mhz transducer and setting a Nyquist limit at 60–80 cm/sec with a frame-rate between 23 and 30 frames/Sect. [30].The images acquired are then analyzed using a dedicated software that provides a visual representation of flow vectors [30]. More recently, the Hyper-Doppler software (Esaote, Genova, Italy, Fig. 2) allows quantitative assessment of the main vortex’s parameters based on the VFM technique [31]. However, VFM is dependent upon high-quality echocardiographic images and adequate color Doppler signal; transverse velocities are only reconstructed with this technique and not actually calculated. Finally, the temporal resolution of the modality can affect the accuracy of vector calculation, particularly at low velocities [32]. Particle imaging velocimetry (PIV) echocardiography exploits the movement of contrast microbubbles to display flow motion within the cardiac chambers [33, 34]. In detail, an algorithm allows the recognition of a specific feature in the region of interest which is subsequently tracked in the next frame; the new identified position is then used for the calculation of both feature displacement and velocity.

**Fig. 3:**
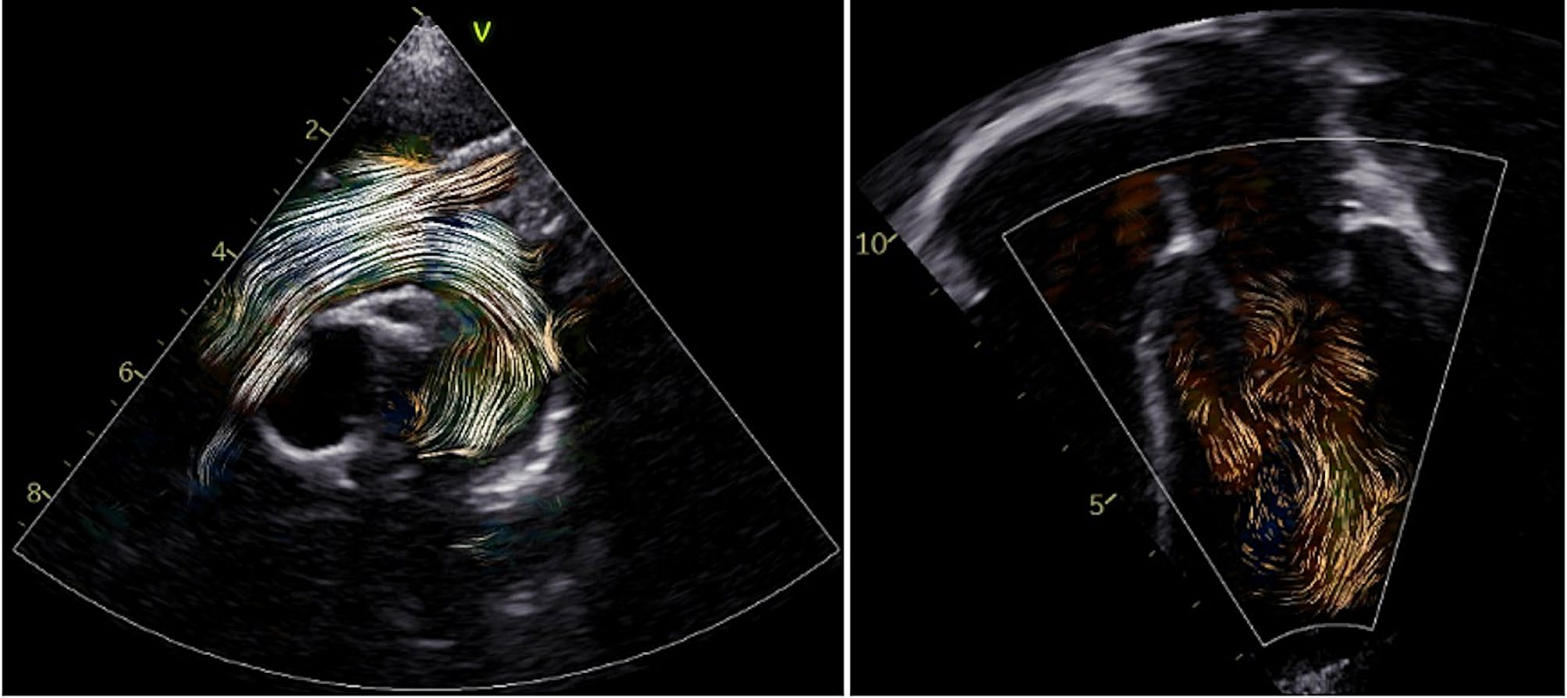
Intracardiac flow analysis using blood speckle tracking echocardiography. Fluid dynamic analysis using blood speckle tracking of the aortic arch (left) and the left ventricle (right) in a healthy child

This technique requires a higher frame rate than VFM, with greatest accuracy estimated from a computational model at 113 frame-per-second [33]. In addition, it is highly dependent on adequate bubble density, as either too dense or too sparse bubbles may hinder accurate feature tracking [33]. Finally, speckle tracking echocardiography (STE) has been applied to fluid dynamics to track the movement of speckles generated by red blood cells with very high temporal resolution [35]. Blood speckle echocardiography is fast, and reproducible and may be used as a complementary tool to color Doppler for a deeper understanding of physiologic and pathologic flow and vortex patterns [36]. This analysis overcomes both the mathematical assumption and the angle dependency of c olor D oppler techniques but, requiring very high frame rates, is currently available only for neonatal/pediatric and transesophageal probes **(**Fig. 3) [37].

### Cardiac magnetic resonance

Cardiac magnetic resonance (CMR) is the gold standard imaging modality for volumes and function assessment and has the unique capability to provide tissue characterization [38]. In addition, two-dimensional (2D) phase-contrast (PC) sequences can be used to assess blood flow within cardiac chambers and major vessels [39, 40]. Using in-plane or through-plane acquisition, this sequence allows for visualization and quantification of flow rates across the chosen structure of interest. Different hemodynamic parameters can be obtained, including forward flow, cardiac output, peak velocities, regurgitant fractions, and amount of shunt flow [39]. However, PC CMR sequences should be carefully planned considering that flow information will be collected only in the selected imaging plane. This implies that if multiple planes are needed, the whole scanning time could be significantly increased. 2D PC-CMR is also sensitive to magnetic field inhomogeneities, presence of arrhythmias and motion artifacts, including cardiac and respiratory movement that can degrade image quality and effect accuracy of flow measurements. Selecting an optimal Velocity encoding (VENC) during image acquisition is also crucial, as VENC set too low may generate artifacts that reduce accuracy of flow measurements [41, 42]. The assumption of laminar flow behind PC sequences data computation is another limitation, particularly in the presence of stenosis or turbulent flow. Some of these limitations have been addressed and overcome by a novel technique. 4D flow MRI provides full volumetric coverage enabling comprehensive flow assessment in three dimensions (3D) over time (4th dimension), reducing the dependency on precise plane selection and improving the understanding of complex flow patterns [43]. 4D flow MRI captures data throughout a 3D volume, allowing the simultaneous assessment of multiple vessels and/or chambers and a retrospective analysis of data already acquired. This flexibility is particularly valuable in clinical settings where new clinical questions or needs may arise after the initial scan. Moreover, the technique allows visualization of vortical flows, undetected by 2D PC MRI. A range of hemodynamic parameters can be in fact obtained including not only velocity and pressure gradients but also flow rate, wall shear stress and vorticity [43, 44]. 4D Flow MRI is a novel imaging technique allowing a time-resolved 3D accurate assessment of flows and main energetic parameters (viscous energy loss, KE dissipation, and turbulent KE) while overcoming many limitations of TTE and PC MRI using a temporal resolution of 30–40 ms and a spatial resolution < 3 mm [43, 45, 46]. Despite all these advantages, 4D flow MRI typically requires longer acquisition time compared to standard 2D PC imaging, which is challenging in severely hill patients with difficult to breath hold. Advances in parallel imaging and compressed sensing as well as artificial intelligence and machine learning may help to overcome this issue in the future [47]. The data acquired usually requires large computational power and large space of storage for both processing analysis and visualization. In addition, the technique requires high specialization and dedicated software for both acquisition and post-processing [43]. All these factors contribute to reduce the availability of the modality and significantly increasing its cost. A comparison of the different imaging techniques used to assess intracardiac fluid dynamics is shown in Table 2.

**Table 2:** Comparison of intracardiac flow visualization techniques

Comparison of intracardiac flow visualization techniques				
	CE-PIV	Color Doppler VFM	CMR	Blood Speckle Imaging
**Signal source**	Tracking of contrast microbubbles	Color Doppler-based flow mapping	Velocity-encoded phase contrast MRI	Tracking the movement of blood speckles
**Resolution**	Good spatial resolution in 2D, limited 3D	Good spatial resolution in 2D, limited 3D	High spatial resolution in 3D and 4D	Good spatial resolution in 2D, limited 3D High temporal resolution
**Advantages**	Bedside Low cost Short process time Accurate vortex visualization Validated quantitative parameters	Bedside Low cost Short process time Not require contrast microbubbles	Unrestricted access Full 3D and 4D capability	Bedside Low cost Short process time Not require contrast agents Free of aliasing artifacts
**Limitations**	Need contrast agent Need high frame rate Acoustic shadowing	Lacking validated parameters Need manual de-aliasing Lower temporal resolution Acoustic shadowing	Need several cardiac cycles Longer examination time Limitation in intracardiac devices No Bedside High cost	Lacking validated parameters Available only for neonatal and pediatric patients and for TEE Underestimation of blood peak velocity Need high frame rate Acoustic shadowing Possible signal dropouts

Legend 2D: two-dimensional; 3D: three-dimensional; 4D: four-dimensional; CE: contrast echocardiography; TEE: trans-esophageal echocardiography; PIV: particle image velocimetry; VFM: vector flow mapping; CMR: cardiac magnetic resonance; LV: left ventricular; RV, right ventricular; LA: left atrium

### Other imaging techniques

There is currently few data on the role of other imaging techniques in evaluating intracardiac fluid dynamic; c omputational fluid dynamics based on cardiac computed tomography (CCT) acquisitions has been recently applied in experimental models for this purpose [48]. Obermeier et al. used end-diastolic and end-systolic CCT frames to reconstruct LV geometries and movement and demonstrated that these models can evaluate LV functional changes in patients with mitral valve [49]. A CT-based assessment of vortex structures within the LA and the LA appendage can help unveiling the mechanism of thrombosis and stroke [23, 25]. Recent advancements in computational tools have significantly enhanced our understanding of cardiovascular physiology, particularly through the use of computational fluid dynamics (CFD) and digital twins of the heart [50]. CFD has allowed for detailed simulations of complex blood flow patterns, enabling precise analysis of hemodynamic parameters in both normal and pathological states [51]. Meanwhile, digital twins—highly detailed virtual models of the heart—integrate anatomical, electrophysiological, and hemodynamic data, providing a comprehensive platform for personalized diagnosis, treatment planning, and the prediction of disease progression [52]. Together, these innovations represent a powerful shift towards more accurate and individualized cardiac care.

### Potential clinical applications

**Fig. 4:**
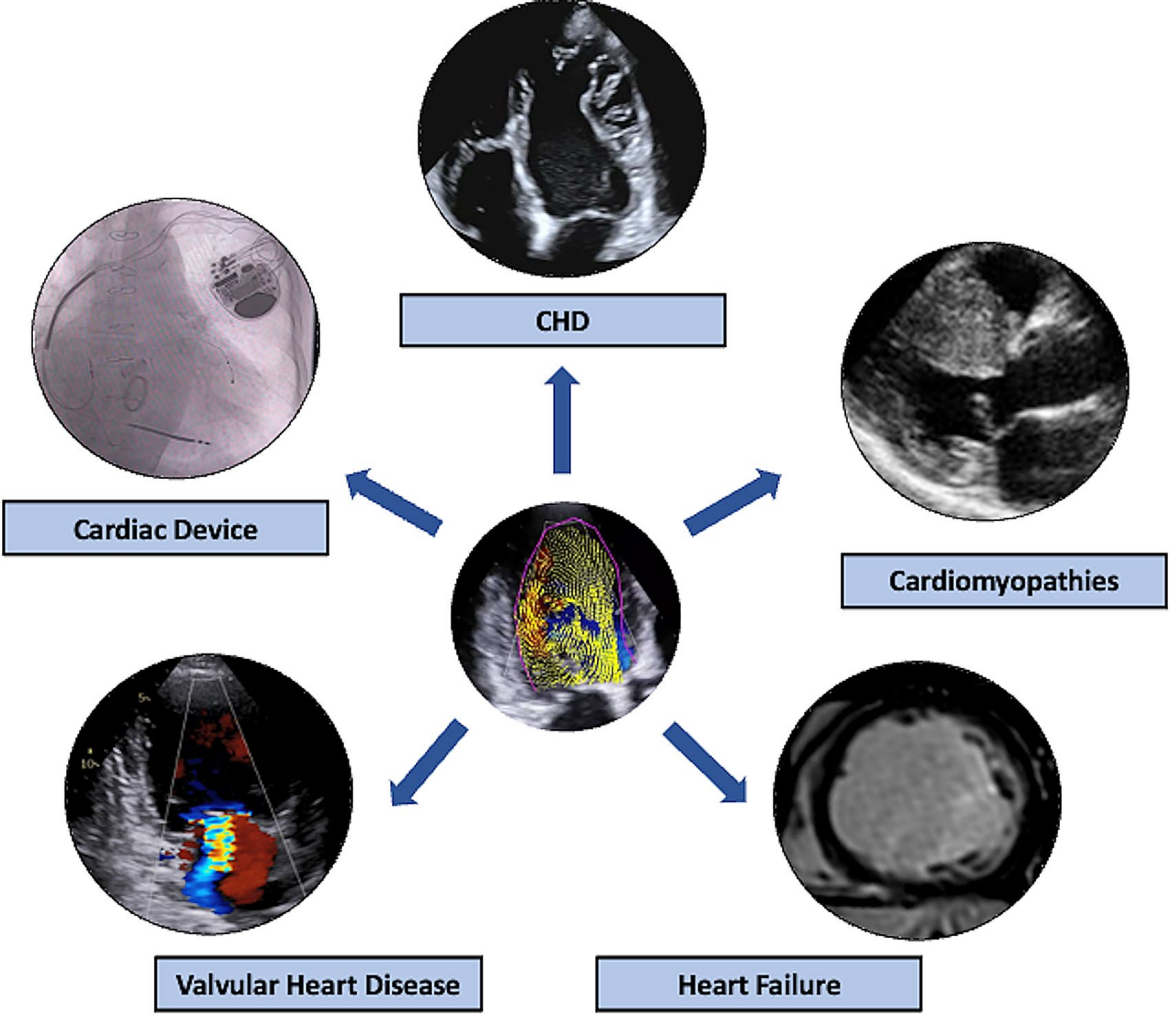
Potential clinical indications of fluid dynamic analysis

Qualitative and quantitative assessment of intracardiac fluid dynamics is nowadays mainly limited to the research setting; however, several studies have investigated the potential role of these novel parameters in different clinical settings (Table 3; Fig. 4).

**Table 3:** Fluid dynamic changes in cardiovascular conditions

Cardiovascular Disease	Fluid dynamic analysis
Dilated Cardiomyopathy (DCM)	Large vortex, characterized by increased vortex width (VW) and reduction of vortex length (VL) Reduced Vortex Depth (VD) Reduced Vortex Strenght (VS) Increased Kinetic Energy (KE) and Kinetic Energy Dissipation (KED)
Advanced Heart Failure (HF) with Left Ventricular assist device (LVAD) implantation	Increased KED Reduced VS Reduced Vortex Area (VA) in case of total LVAD support Increased VS and VA in case of partial LVAD support
Hypertrophic Cardiomyopathy (HCM)	Increased diastolic KED Reduced VFT time index in non-obstructive HCM Increased VFT in obstructive HCM
Athlete’s Heart	Increased VFT, VA and KED No significant differences in VD compared with normal sedentary subjects
Cardiac Resynchronization Therapy (CRT)	Longitudinal alignment of hemodynamic forces along the LV major longitudinal axis in CRT responders patients Increased KED, VA and vorticity fluctuation in CRT non-responders patients or during CRT deactivation
Mitral Valve (MV) diseases	MV stenosis: Higher Vortex Formation Time (VFT) MV regurgitation: Reduced vortex velocity Increased peak velocity and vortex vorticity, Increased asymmetric spatial properties
Mitral valve intervention	Mitral prosthetic valve: Formation of an asymmetric large counterclockwise vortex with increased KED in patients with mitral prosthetic valve Mitraclip Implantation: Reduced peak velocity and vortex vorticity, Formation of two different jets with lower velocity, shear layers and chaotic flow
Aortic valve (AoV) diseases	AoV stenosis: Increased VA, VL and VD Large vortex, displaced towards LV apex AoV regurgitation: Alteration of vortex formation with increased KED This is also present in case of paravalvular leak after transcatheter aortic valve replacement
Tetralogy of Fallot (ToF)	Unrepaired ToF: Reduced KED and increased biventricular flow rotation intensities Repaired ToF: Reduced LV peak systolic KE and increased RV diastolic peak KE, with KE mainly located in the pulmonary regurgitation volume Increased of KE in patients with non-restrictive physiology After surgical pulmonary valve repair: KE values in both ventricles are restored
Transposition of Great Arteries	Loss of spiral flow pattern and presence of laminar flow pattern After Fontan operation: Short, wide and round vortex Increased of KED and vortex vorticity

### Heart failure

Dilated cardiomyopathy (DCM) is characterized by progressive LV dilation, geometric remodeling and impaired systolic function [53]. The transition from a normal ellipsoidal shape to a more spherical configuration results in a larger vortex, that persists longer during diastole and is displaced more toward the apex. However, A 4D-flow in vivo study demonstrated that despite being larger, the vortex rings may not reach the endocardial surface of a very dilated heart, with resulting increase of blood stasis and risk of thrombus formation [54]. In dilated hearts the vortex occupies only about 20% of the LV diastatic volume compared to the 50% of normal hearts [54]. Vortex in DCM patients also presents reduced strength compared to healthy controls [55]. The kinetic energy (KE) is instead higher, with faster KE dissipation [31, 56–59]. In detail, DCM patients exhibit a characteristic biphasic vortex behavior during diastole with a late diastolic boosting corresponding to atrial contraction [58]. Eriksson et al. [60] demonstrated that, under equal stroke volume conditions, the proportion of LV inflow that is directly ejected into the outflow is significantly smaller in DCM patients. They also have high KE per ml of residual end-diastolic volume, likely reflecting the impact on fluid dynamics of a less compliant cardiomyopathic myocardium [60].

### Left ventricular assist device (LVAD)

In the context of advanced heart failure, a left ventricular assist device (LVAD) is a critical option for mechanical circulatory support, either as bridge to cardiac transplantation or as destination therapy [61]. As already mentioned, a severe impairment of LV function is associated with the disruption of vortex patterns, with consequent increased energy dissipation and cardiac workload [62].

The presence of the inflow cannula into the LV apex and the outflow cannula in the ascending aorta, along with the pulsatile nature of the LVAD flow, further alters the formation and pattern of cardiac vortices [63]. The presence of LVAD influences vortex strength, as at low LVAD flows the strength of the vortex decreases as the result of a decrease in the core area. At higher flows there is an initial increase in vortex strength compared to unassisted DCM; however, this quickly disappears likely as a consequence of LVAD apical flow washing out vortex rings [55]. Differences were also noted between partial and full LVAD support; in fact, under partial LVAD support, the intraventricular vortex increased significantly in both strength and dimension. The duration of vortex formation also extended from 0.1 s in heart failure to 0.25 s [64]. All these changes may improve blood mixing, reduce thrombotic risk and lower cardiac workload, potentially aiding in heart function recovery. In contrast, with full LVAD support, no vortex was detected throughout the cardiac cycle due to continuous blood flow, highlighting a more physiological fluid dynamic pattern with partial support model [64].

Pulsatile-flow LVADs are associated with a greater likelihood of myocardial recovery and improved renal function but face also several design challenges, including lower biocompatibility, inefficient hydraulic performance in smaller sizes, and a higher risk of thrombosis and hemolysis [65]. These issues primarily arise from the complex flow states within the blood chamber, which have been a significant focus in the LVADs technological development. A computational fluid dynamics model validated with echo-PIV demonstrated that a device with an asymmetric channel arrangement and a 45-degree intersection between the inflow and outflow channels was more able to mimic LV geometry and obtained a continuous vortex flow that closely approximate the intraventricular physiological flow [66].

Vortex analysis can be therefore useful to optimize LVAD application, with the aim to obtain intraventricular flow patterns as similar as possible to native ventricles, thereby potentially reducing some of the related complications.

### Cardiac resynchronization therapy (CRT)

The application of vortex analysis could be useful to guide the implantation and optimization of cardiac resynchronization therapy (CRT). Studies using the Echo-PIV technique revealed that, in subjects responding to active pacing there is a longitudinal alignment of hemodynamic forces along the LV major longitudinal axis, as in healthy hearts [67, 68]. Discontinuation of pacing results in dissipation of this alignment, accompanied by an increase of transverse components that lacks of propulsive function [57, 67]. This is not observed in CRT non-responders, where intraventricular flow fails to align during CRT activation [69, 70]. For instance, parameters such as energy dissipation, vortex area, and vorticity fluctuation were found to be elevated in CRT non-responders and during CRT deactivation [70], while during CRT activation these parameters increase only in CRT non-responders and are associated to poor ventricular synchronization [70, 71].

### Valvular heart disease (VHD)

Given its key role in vortex formation, abnormalities of the mitral valve may significantly impact intracardiac fluid dynamics. In experimental studies, even a brief and moderate rise in LV afterload can reduce transmitral flow efficiency, resulting in VFT values moving away from their optimal range [72]. This has been later demonstrated in vivo in patients with different degree of mitral stenosis; VFT was in fact suboptimal (higher) in patients with severe mitral stenosis, correlating with both LA volume index and mean transmitral pressure gradient. In case of mitral regurgitation, vortices present slower velocity, delays in peak velocities, and asymmetric spatial properties [73].

The presence of a mitral prosthetic valve both alters left ventricular vortex formation and increases KE dissipation [74]. In the presence of a mechanical mitral prosthesis the blood is directed towards the interventricular septum, with an asymmetric growth of a large counterclockwise vortex that moves away from the LV outflow tract and creates an unfavorable energetic model [62]. Similarly, an experimental model demonstrated that changing the orientation of mitral bioprosthesis from a standard position to an angled one with a 15˚ tilt toward the LV septum, resulted in a larger counterclockwise vortex [75].This redirects the blood away from the LVOT, with greater energy expenditure to maintain forward flow in the aorta and increased turbulence extending into the LVOT [61]. While MitraClip transcatheter edge-to-edge repair is effective in reducing the degree of mitral regurgitation, this significantly affects left ventricular flows as demonstrated in an in vitro LV simulator of a porcine MV [76]. Significant mitral regurgitation caused higher peak velocity and vorticity, that reduced after MitraClip implantation [76]. However, after the procedure, there was evidence of two different jets with lower velocity, shear layers and chaotic flow [76]. Understanding fluid dynamics changes in case of MV repair may contribute to optimize the use of these devices and improve clinical outcomes [75, 77, 78]. A recent study using Hyperdoppler analysis to quantify intracardiac flow changes in patients with severe aortic stenosis (AS) demonstrated a significant change in vortex localization, vorticity, and vortex energy parameters in these patients compared to healthy controls [79]. Patients with severe AS had in fact greater vortex area, length and depth and increased vortex intensity resulting in a larger vortex, displaced towards the LV apex, similarly to what was observed in DCM patients [79]. Vortex depth was also able to differentiate with good accuracy patients with AS from both healthy controls and patients with concentric LV remodeling and no AS [79]. Patients with low-flow low-gradient AS had also similar intracardiac fluid dynamics of patients with normal-flow high-gradient AS, both in localization and energy parameters [79].

The regurgitant jet in case of aortic regurgitation, hinders vortex formation with increased energy dissipations [80]. This is true also in the presence of paravalvular leak after transcatheter aortic valve replacement, with posterior paravalvular leaks having a more negative impact on intracardiac fluid dynamic compared to anterior ones [81].

### Congenital heart disease (CHD)

The evaluation of RV geometry and function plays a crucial role in the assessment of clinical outcomes and long-term survival in both adults and children with congenital heart diseases (CHD) [82–84]. This is often challenging with conventional TTE, and fluid dynamics analysis can provide more insight into the diagnosis and management of this heterogeneous group of patients. While advancements in therapeutic management improved survival rates of patients with Tetralogy of Fallot (ToF), significant pulmonary regurgitation (PR) and RV dilatation are frequently observed long-term complications after repair [85, 86]. An echo-PIV study demonstrated lower KE dissipation and higher biventricular flow rotation intensities in patients with TOF compared to controls, changes likely related to ventricular dilatation and reduced ventricular compliance [87]. Patients with repaired TOF exhibit also reduced RV direct flow which correlates with both RV remodeling and exercise capacity [88].They also demonstrated lower LV peak systolic KE and higher RV diastolic peak KE, with KE mainly located in the pulmonary regurgitation volume [88, 89]. Interestingly, all these patients had normal LV systolic function; in addition, a higher increase in KE was noted in patients with non-restrictive physiology [89]. After surgical pulmonary valve repair physiological KE values were restored in both ventricles [88]. A helical flow pattern is observed in large arteries, where has the role to ensure a more uniform distribution of endothelial shear stress [90]. This is the result of both the looping of the great arteries during cardiac development and the curvature of the aortic arch [90]. Riesenkampff et al. [91] demonstrated that this spiral pattern is lost in patients with transposition of the great arteries (TGA), a congenital defect characterized by anatomic reversal of the aorta and pulmonary artery, originating respectively from the morphologic RV and LV [85]. The use of blood speckle imaging to evaluate flow patterns proved valuable in identifying the source of increased gradients observed on Doppler analysis in a complex TGA case. The images revealed a laminar flow across the unobstructed pulmonary valve, with no turbulence or vortex formation, originating instead from a large associated ventricular septal defect [37].

Fontan operation improves outcome of patients with single-ventricle physiology, but is associated with long-term complications such as arrhythmias, heart failure, and liver disease, necessitating lifelong monitoring and management [92]. Echo-PIV was feasible in these patients and demonstrated abnormal intracardiac flow patterns with significantly shorter, wider and rounder vortex compared to normal hearts [93]. Changes in intracardiac hemodynamic may be linked to decreased exercise capacity in Fontan patients. A 4D flow CMR study showed that KE, viscous energy loss and vorticity increase during dobutamine infusion and had a negative correlation with exercise capacity measured by maximum oxygen uptake [94]. Total extracardiac cavo-pulmonary connection (TCPC) is a surgical technique used to connect the inferior vena cava to the pulmonary arteries in patients with single-ventricle physiology; the left-sided version of this surgery promotes a central vortex formation with improved flow efficiency compared to the directly-opposed TCPC which is instead characterized by the absence of vortical structures [95].

### Hypertrophic cardiomyopathy

Patients with nonobstructive hypertrophic cardiomyopathy (HCM) present advanced diastolic dysfunction, and the analysis of vortices exhibits higher diastolic KE dissipation and lower vortex formation time index than health subjects [96, 97]. In case of obstructive HCM, vortex flow evaluation could be helpful to identify systolic anterior movement of the MV leaflet, accompanied by increased vortex formation time [98].

In addition, the amount of filling volume entering the LV in patients with HCM was significantly lower compared to both healthy controls and DCM patients, demonstrating detrimental effects of LV increased stiffness in vortex formation [99]. Differences in VFT index were also found in patients with different patterns of transmitral diastolic filling even in the presence of similar mitral annulus recoil during diastole [100].

It is interesting to highlight that the increase in vortex formation time has been described also in healthy athletes’ hearts, with higher values even when compared with patients with hypertrophic cardiomyopathy [101]. Athletes showed higher values of vortex area compared with normal sedentary subjects, with no significant difference in Vortex depth. Also KED was significantly increased in athletes compared to sedentary controls [31]. This probably relates to the higher flow velocities of early LV filling, the supernormal diastolic function and physiological adaptations of the LV in thesesubjects [101, 102].

### Myocardial infarction

Changes in intracardiac fluid dynamics are part of the functional alterations that have been observed in patients experiencing acute myocardial infarction (MI). It has been in fact demonstrated that acute MI patients have lower peak-wave KE than controls, that is indicative of an early loss of diastolic function [103]. Garg et al. also demonstrated that systolic KE is decreased in MI patients compared to controls [104]. The extent of myocardial damage linearly correlates with the reduction in vortex formation time and vortex sphericity index [105]. Further studies underlined that systolic KE significantly decreases over the first 3 months from the acute event, suggesting that this could provide an optimal timeframe for rehabilitation [106]. Analyzing intraventricular vortex flow may help assessing the risk of thrombus formation in this subset of patients; a reduced vortex depth and relative strength were in fact significantly associated with the development of apical thrombus in a cohort of 57 patients with acute anterior MI [107].

## Conclusions

While analysis of intracardiac flows has deepened our understanding of cardiac fluid mechanics in both in vitro and in vivo models, its integration into routine clinical practice remains limited. Most of the available imaging techniques are in fact costly, time-consuming, and not widely available; in addition, they require specialized software and expertise in both acquisition and post-processing which further limit their accessibility. The interpretation of changes in vortex pattern is also challenging due to heterogeneity among patients and lack of established guidelines on how to integrate this assessment into clinical decision-making. Finally, the exact clinical implications of these findings remain incompletely understood. For all these reasonsthe role of the vortex remains to date largely theoretical; however, the increase in awareness regarding their potential role along with the growing number of studies exploring this field and the efforts in developing novel and more accessible techniques could be useful to overcome this gap in the future, making the assessment of intraventricular flow dynamics a promising imaging marker for several clinical settings.

## Data Availability

No datasets were generated or analysed during the current study.

## Abbreviations

2D: Two dimensional
3D: Three dimensional
4D: Four dimensional
AF: Atrial fibrillation
AS: Aortic stenosis
CHD: Congenital heart disease
CFD: Computational fluid dynamics
CMR: Cardiac magnetic resonance
CRT: Cardiac resynchronization therapy
CV: Cardiovascular
DCM: Dilated cardiomyopathy
EF: Ejection fraction
HCM: Hypertrophic cardiomyopathy
KE: Kinetic energy
KED: Kinetic energy dissipation
LA: Left atrium
LV: Left ventricle
LVAD: Left ventricular assist device
LVOT: Left ventricular outflow trait
MI: Myocardial infarction
MV: Mitral valve
PC: Phase-contrast
PIV: Particle imaging velocimetry
PR: Pulmonary regurgitation
RV: Right ventricle
STE: Speckle-tracking echocardiography
TCPC: Total extracardiac cavo-pulmonary connection
TGA: Transposition of great arteries
TOF: Tetralogy of Fallot
TTE: Transthoracic echocardiography
VENC: Velocity encoding
VFM: Vector flow mapping
VHD: Valvular heart disease
